# First person – Masayoshi Ko

**DOI:** 10.1242/dmm.048973

**Published:** 2021-03-28

## Abstract

First Person is a series of interviews with the first authors of a selection of papers published in Disease Models & Mechanisms, helping early-career researchers promote themselves alongside their papers. Masayoshi Ko is first author on ‘ Modulation of serotonin in the gut-liver neural axis ameliorates the fatty and fibrotic changes in non-alcoholic fatty liver’, published in DMM. Masayoshi is an MD and PhD student in the lab of Kenya Kamimura and Shuji Terai at Niigata University, Japan, investigating the involvement of multi-organ linkage via autonomic nerves in non-alcoholic fatty liver (NAFLD).


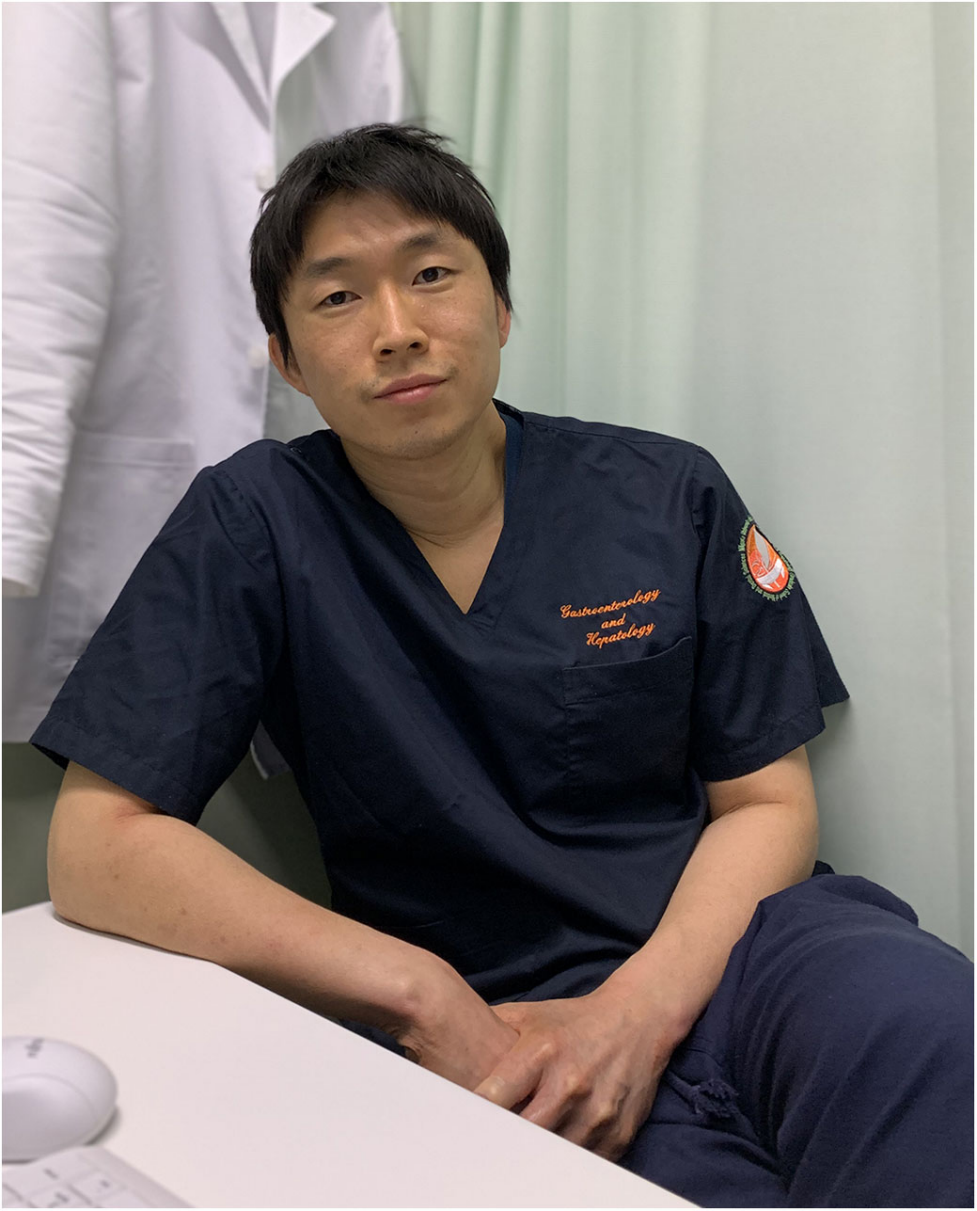


**Masayoshi Ko**

**How would you explain the main findings of your paper to non-scientific family and friends?**

NAFLD is a chronic liver disease mainly triggered by lifestyle-related causes. With an increasingly obese population, the number of cases of liver cancer caused by NAFLD has been increasing. The pathophysiology of NAFLD remains unclear and, as a consequence, there is still no definitive treatment against it. In order to clarify the involvement of autonomic nervous system signalling in NAFLD, NAFLD model mice were divided into two groups, with and without blocking of afferent visceral nerves from the liver. With this method, we aimed to compare the progression of NAFLD over time and to evaluate the intestinal tight junction protein expression, intestinal flora and small intestinal serotonin. To elucidate the involvement of serotonin in detail, we have also evaluated NAFLD and intestinal tight junction protein in mice fed with serotonin receptor antagonists. By blocking the afferent visceral nerve, we have observed suppression of the expression of serotonin in the small intestine, the increase in expression of intestinal tight junction proteins, strengthening of barrier function and suppression of NAFLD progression. The intestinal flora also changed over time. After analysis of these results, it was inferred that the autonomic nervous pathway was involved in the pathological progression of NAFLD, and serotonin was identified as one of its effectors. This result was also reproduced by administrating serotonin receptor antagonists to NAFLD model mice. Our study, which was focused on the pathophysiology of NAFLD, revealed that expression of serotonin in the small intestine by the gut-liver neural axis via the autonomic nerve pathway lowers the intestinal barrier function and promotes NAFLD.

“With an increasingly obese population, the number of cases of liver cancer caused by NAFLD has been increasing.”

**What are the main advantages and drawbacks of the model system you have used as it relates to the disease you are investigating?**

Mice fed with a methionine and choline-deficient (MCD) diet are often used as a model for NAFLD. The MCD diet prevents the synthesis of lipoproteins that transport lipids from the liver to other tissues, causing fat to accumulate in the liver and provoke NAFLD. However, the MCD diet can cause fatal weight loss. In this study then, we have used mice fed with the choline-deficient defined L-amino-acid (CDAA) diet or with a high-fat diet (HFD) as a model for NAFLD. The CDAA diet is similar to a the MCD diet with additional methionine, inducing only a mild and non-lethal weight loss. The advantage of the CDAA diet is that it is not lethal and allows mice to develop NAFLD in a short period of time. However, the CDAA diet differs from the natural course of human NAFLD development. On the contrary, the HFD takes longer to develop NAFLD but this is similar to the course of human NAFLD development.

**What has surprised you the most while conducting your research?**

During our research, we were surprised by the consequences of the blockage of only one autonomic nerve: it changed the intestinal flora, strengthened the intestinal barrier and improved fat deposition and fibrosis in the liver. We were also able to confirm that multi-organ linkage via autonomic nerves plays an important role in maintaining biological functions.

**Figure:**
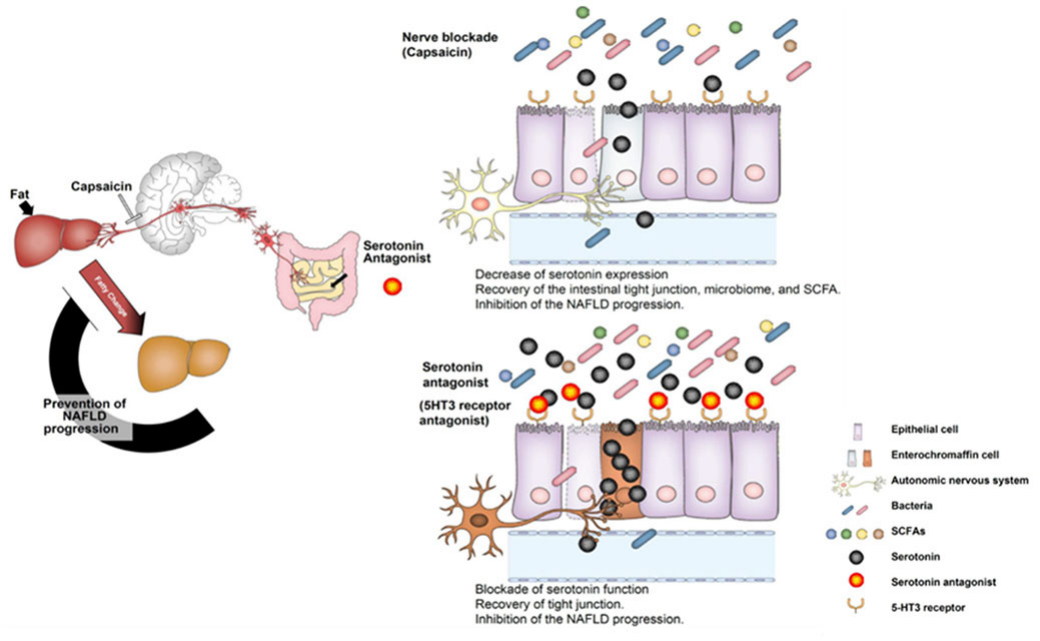
**The gut-liver axis via the autonomic nervous pathway controls the release of serotonin in the small intestine. Serotonin reduces intestinal barrier function and promotes NAFLD.**

**Describe what you think is the most significant challenge impacting your research at this time and how will this be addressed over the next 10 years?**

From this study, it was found that serotonin affects NAFLD, although the infiltration of inflammatory cells in the liver has not been yet evaluated. In addition, it was found that, by blocking the autonomic nerves, the intestinal flora and short-chain fatty acids were changed. However, the relationship between the short-chain fatty acids and the intestinal flora is unknown. This leaves some unanswered questions that need to be further considered. An additional study would be needed to evaluate the influence of the administration of serotonin antagonist on intestinal bacteria. We believe that continuing this research will lead not only to a further elucidation of the pathophysiology of NAFLD, but also to the identification and development of new treatments against NAFLD.

**What changes do you think could improve the professional lives of early-career scientists?**

In Japan, it is challenging for doctors to carry out basic research, such as using animal models, while working in secondary care other than a university hospital. I believe that our research would be more easily carried out if a suitable research environment could be provided.

**What's next for you?**

As a gastroenterologist, my goal is to improve my knowledge, learn new procedures and treat as many patients as possible. My immediate goal is to obtain a specialist qualification in gastroenterology, and the goal of this research is to aid the development of new treatments that may help patients in my speciality.

**Anything you would like to say?**

I would like to thank Kenya Kamimura and Professor Shuji Terai in the Division of Gastroenterology and Hepatology at the Niigata University for their continued guidance. I would also like to thank Takashi Owaki, Takuro Nagoya, Norihiro Sakai, Itsuo Nagayama, Yusuke Niwa, Osamu Shibata, Chiyumi Oda, Shinichi Morita, Atsushi Kimura, Ryosuke Inoue, Toru Setsu, Akira Sakamaki and Takeshi Yokoo in the Division of Gastroenterology and Hepatology at Niigata University for cooperating with my research. I would also like to acknowledge Takao Tsuchida for his excellent assistance with histological analyses, and thank Nobuyoshi Fujisawa, Kanako Oda, Shuko Adachi, Toshikuni Sasaoka and all staff members at the Division of Laboratory Animal Resources at Niigata University.
